# The effect of educational intervention according to mindfulness on the psychological well-being of female heads of households

**DOI:** 10.1186/s12905-024-03125-9

**Published:** 2024-06-03

**Authors:** Fataneh Mahmoudi, Maryam Zarnaghash, Nadere Sohrabi Shegefti, Majid Barzegar

**Affiliations:** 1 Educational Psychology, Marvdasht Branch, Islamic Azad University, Marvdasht, Iran; 2 Department of Psychology, Marvdasht Branch, Islamic Azad University, Marvdasht, Iran; 3 Department of General Psychology, Marvdasht Branch, Islamic Azad University, Marvdasht, Iran; 4 Department of Educational Psychology, Marvdasht Branch, Islamic Azad University, Marvdasht, Iran

**Keywords:** Educational intervention, Mindfulness, Psychological well-being, Women as the heads of household

## Abstract

**Objective:**

The low level of psychological well-being is one of the most common problems of the women who are the heads of households, and various educational programs have been conducted to improve the psychological well-being of such a group. The present study was conducted to outline the effectiveness of mindfulness-based educational intervention in the psychological well-being of women as the heads of households.

**Methods:**

This research as a semi-experimental study was done by pretest–posttest design and control group. The study statistical population included all women as the heads of households in Shiraz, who had a file in the Shiraz based welfare office and at the same time their children were studying in schools covered by the 2nd district educational department of Shiraz in 2022–2023. Out of the female heads of the households in the statistical community, 30 women were selected as Purposive sampling and then randomly divided into two 15-individual groups, including experimental group (training on mindfulness-based therapy) and control group. The research tool included Ryff’s psychological well-being scale (Ryff, 1989). The experimental group was subjected to training on mindfulness-based therapy (Baer et al., 2006) during eight 90-min sessions. However, the control group did not receive any intervention.

**Result:**

After running the intervention, a significant difference was seen between the components of autonomy, personal growth, purpose in life, self-acceptance, positive relationship with others, and psychological well-being after running the educational intervention between the experimental and control groups (*P <* 0.05), while no meaningful difference popped up between the experimental and control groups in terms of environmental mastery as one of the aspects measured in psychological well-being scale after the educational intervention (*P* = 0.602).

**Conclusion:**

According to the results, it seems that psychologists can benefit from the method of training on mindfulness-based therapy in order to increase the psychological well-being of women as the heads of households.

## Introduction

As defined by the World Health Organization, female heads of the households refer to the women who are in charge of providing the material and spiritual needs of their family [1]. Pursuant to the definition of the Iranian Statistics Center, the women who head their family without the regular presence or support of an adult man and who are responsible for the economic and social management of the family are called female heads of the household [2]. In Iran, the number of such women is increasing day by day so that it was reported as (7.3%) in 1986, (8.4%) in 1996, (9.4%) in 2006, (12.1) in 2011, and (12.7) in 2016 [2]. Most of the female heads of the households are challenging with poverty and disability to afford the economic affairs of the family, the problems which lead to decreasing efficiency and mental health in such women [3–5]. So that compared to other women, the examination of the physical and mental health status of such women has revealed that this group has less physical and mental health and is exposed to all kinds of physical and mental diseases [6].

This group of women has displayed higher levels of vulnerability and stress, lower potential to solve their problems, and feeling more guilty, depressed, anxious, and worried than the women not being the heads of the households [7–9].

In addition to the above mentioned cases, the problems of this group of women result in psychological nuisances and subsequently, their psychological well-being declines [10, 11]. Psychological well-being is one of the concepts concentrated by positive psychology and defined as optimal performance and it empowers a person to adopt acceptable methods to solve problems when exposed to various sorts of pressures in life [12].

Ryff (1989) put psychological well-being (PWB) as striving for perfection to realize one's true potential, which measures six aspects: autonomy, personal growth, positive relationship with others, purpose in life,self-acceptance, and environmental mastery [13].

Increasing the psychological well-being of the women as the household heads leads to such group’s quality of life getting improved, their psychological distress getting decreased, being correctly evaluated and really recognized, and increased power of coping with hardships [14]. Considering the above cases, we can firmly claim that implementing the due interventions to promote psychological well-being is extremely critical [15], one of which is mindfulnes-based intervention. Mindfulness affects the development of non-judgment, intentional awareness, and paying attention to the present moment, which enables focused attention on the present moment, processing all aspects of immediate experience, including cognitive, physiological, or behavioral activities [16]. Mindfulness helps a person to percieve this point that negative emotions may occur, but they are not a fixed and permanent part of the personality. Besides, it allows a person to respond to events with thought and reflection, rather than responding involuntarily and thoughtlessly [17] and finally, it brings about changes in the person's view of him/herself [18].

The results of various studies indicated the positive impact of mindfulness-based interventions on promoting psychological well-being. As reported by the study conducted by Keshmiri et al. (2019), mindfulness plays a moderating role in psychological well-being and its based intervention, resulting in psychological well-being’s improvement [19]. The results of another study done by Elliot et al. (2019) displayed a direct relationship between mindfulness and psychological well-being [20].

Regarding this matter that women are mentioned as the skeleton of social development and the main player of family health and are in charge of very demanding roles and responsibilities, the necessity of which is being fully physically and mentally healthy, and more important than that the female heads of the households undergo a high level of psychological problems such as stress and anxiety regarding their special conditions. Such women experience negative emotions due to being exposed to different stressful conditions because of facing problems and challenges in their lives and also as a result of not being adequately aware how to manage and cope with stress, they are under the influence of its negative aspects. They adopt diverse strategies under stressful conditions; Some of them boost negative emotions by representing them as too unpleasant, and some others manage and regulate emotions through acceptance and positive evaluation. Regarding the above cases, it’s highly critical to identify feelings and emotions. According to the issues raised about the female heads of the households, it is necessary to analyze the problems of this group of women, especially in the psychological field, and accordingly, make appropriate decisions for this special group.

One of the novel methods that helps to be in the present moment and identify feelings and emotions and ultimately, to manage and control them is mindfulness. This way that implementing mindfulness-based interventions results in increasing the improvement of the psychological well-being of this group. Since no similar study has been done so far in Fars Province, the present research pursues the goal to illustrate the effectiveness of mindfulness-based intervention in promoting the psychological well-being of the women heading the household in Shiraz, Fras Province.

If the results indicate the positive effect of mindfulness-based intervention on the psychological well-being, we can propose this intervention to be included as the assistive, supportive and rehabilitative interventions group so that to be employed along with other psychosocial supports to promote the quality of life and the potential and coping capacity of the female heads of the households. Moreover, it can be suggested to hold mindfulness-based training workshops for such individuals and their families in order to empower them and this way, to prevent or reduce the due problems and consequences of this group.

## Material and methods

### Study design and participants

The current study as a semi-experimental research was performed using pretest–posttest design and control group. The statistical population of the research included all women as the heads of the households in Shiraz, who had a file in the Shiraz based welfare office and at the same time their children were studying in schools covered by the 2nd district educational department of Shiraz in 2022–2023. This manner that after coordinating with the 2^nd^ district educational department management of Shiraz, all girl and boy primary schools of this district were included in the list and then, they were randomly sampled (the Martyrs of SA Iran Girls’ School and the Martyrs of SA Iran Boys’ School). The inclusion criteria were willingness to participate in the study, being in the age range of 20 to 50 yrs., low mean score of psychological well-being (less than 36) and not receiving simultaneous treatment interventions. And the exclusion criteria included not willing to cooperate at any time of the study, not participating in at least two training sessions, suffering from chronic physical and psychological diseases (according to the information recorded in the file of the female heads of the households), and taking psychiatric drugs.

### Sample size and sampling method

The number of the required samples was 30 women according to the same study done by Sedghi et al. (2019) targeting to show the effectiveness of mindfulness-based training on the psychological well-being and resiliency of female heads of the households [21] and according to Cohen’s Furmula sample size [22], the sample size for each group was 15 subjects.

$${\text{N}}=\frac{({\text{r}}+1){({{\text{Z}}}_{\frac{\upsigma }{2}}+{{\text{z}}}_{1-\upbeta })}^{2}{\upsigma }^{2}}{{{\text{rd}}}^{2}}=\frac{(1+1){(1.96+0.84)}^{2}{1.51}^{2}}{(1){(1.1}^{2})}=29.54\sim 30$$

r = n1/n2—the ratio of sample size:1

σ—pooled standard deviation: 1.51

d—difference of means of 2 groups: 1.1

Z_1-β_—0.84 for power 0.80

Z_α/2_ -1.96 for alpha 0.05

Out of the female heads of the households in the statistical community, 30 women were selected as Purposive sampling and then randomly divided into two 15-individual groups,including experimental group (training on mindfulness-based therapy) and control group.

### Intervention

In order to design the intervention, after obtaining the necessary permission from Shiraz based welfare office and at the same time, the Ryff's Psychological Well-Being Scale (1989) was run among all participants selected as the sample size (30 women), then 15 of them were randomly considered as the intervention group. So, for designing the intervention, the experimental group was subjected to training on mindfulness-based therapy (Baer et al., 2006) during eight 90-min sessions [23]. However, the control group did not receive any intervention. Finally, in the 2th month after completing the intervention, the Ryff’s Psychological Well-Being Scale (1989) was collected again from the female heads of the households. Table 1 depicts the educational intervention program.

**Table 1:** The educational intervention program

Session	Objective	Subject	Time duration (min)	Training method	Educator
1^st^	The purpose of educational sessions	Introducing the group members with each other and with the psychologist Expressing the objectives Communicating, building trust	90	Lectures and Q&A^a^	Expert group and researcher
2^nd^	Introducing role-cognitive well-being and its importance	Definition of psychological well-being, its importance in improving satisfaction and quality of life	90	Lectures, group discussions, and Q&A	Psychologist and researcher
3^rd^ & 4^th^	Definition of mindfulness and its components	Definition of mindfulness, self-awareness based on mindfulness, mindful breathing, making the members familiar with self-awareness skills, knowing the dimensions of self-awareness, introducing emotional self-awareness, correct self-evaluation, understanding one's strengths and weaknesses and one's limitations. Getting to know the skill of empathy and its dimensions, observation, description, non-judgment, and conscious action toward empathy	90	Educational videos and posters	Psychologist and researcher
5^th^	Education based on the components of observation, description, non-judgment, conscious action toward empathy	Defining communication and examining its importance in life, recognizing strong and superficial communication, different communication styles, multiple skills of mindfulness in effective communication, non-judgmental attitude, observing verbal and non-verbal components of the other party, conscious action	90	Lectures, group discussions, and Q&A	Researcher
6^th^	Education based on the components of observation, description, non-judgment, conscious action toward empathy	Emotional experience and types of emotions, the introduction of the physical and cognitive dimensions of emotions, reviewing the emotional experiences of the members and identifying their physical and cognitive dimensions, creating an emotional experience with mental imagery, body scanning, focusing on the mental and cognitive dimensions of emotions, mindful breathing, and relaxation	90	Lectures, group discussions, and Q&A	Researcher
7^th^	Education based on the components of observation, description, non-judgment, conscious action toward empathy	Acquaintance with the skill of controlling and managing stress in mindfulness, practical exercises to focus on the physical components of stress, body inspection, mindfulness training in normal daily stressful activities, mindful breathing with a focus on the components of stress, positive self-talk	90	Lectures, group discussions, and Q&A	Researcher
8^th^	Reviewing the training based on the components of observation, description, non-judgment, conscious action toward empathy	Reviewing the learned materials, expressing the participants’ experiences of applying the learned materials in life	90	Lectures, group discussions, and Q&A	Researcher

^a^Question & Answer

### Data analysis

The data were analyzed using SPSS-24, and the data normality was first measured by the Kolmogorov–Smirnov test. The frequency, mean, and standard deviation were also used to describe the data, and univariate covariance analysis, independent sample t-test, and chi-square test were employed for data analysis. The significance level in all tests was considered 0.05.

### Data collecting questionnaires

1. Demographic Questionnaire: This questionnaire included information such as age, marital status, employment status, and education.
2. Ryff's Psychological Well-Being Scale (1989): This questionnaire developed by Ryff (1989) measures 6 aspects of psychological well-being and happiness (13). Its shortened form is an 18-item questionnaire measuring 6 subscales: autonomy, personal growth, positive relationship with others, purpose in life, self-acceptance, and environmental mastery. The scoring of this questionnaire is based on a 6-point Likert scale (1 = Absolutely Disagree to 6 = Absolutely Agree). Items 1, 3, 4, 5, 9, 10, 13, and 17 are the reverse-scored ones. The minimum score is 18 and the maximum score is 108. In Iran, the research done by Khanjani aimed to investigate the factor structure and psychometric properties of the short form of Ryff’s psychological well-being scale in college students reported the internal consistency of the above sclale’s factors using Cronbach’s alpha of 0.71% [24].

## Results

The demographic and background information of the participants is given in Table 2. The mean and standard deviation of age in the intervention and control groups were 44.87 ± 5.71 and 44.73 ± 4.46 yrs, respectively. Most of the participants in both groups were widows, with high school/diploma education and housewives. The Chi-square test displayed no significant difference between the experimental and control groups in terms of education (*P =* 0.33), occupation (*P =* 0.69), and marital status (*P* = 0.67). In addition, the t-test showed no meaningful difference between the two groups in terms of age (*P =* 0.94) (Table 2).

**Table 2:** The demographic and background information of the participants in both groups

Variable		Experimental group (%)	Control group (%)	*P*-value
**Age**		44.87 ± 5.71	44.73 ± 4.46	0.94^*^
**Marital status**	married	2(13.3)	4(26.7)	0.67^**^
	Separated	5(33.3)	4(26.7)	
	widow	4(26.7)	5(33.3)	
	Others (fugitive spouse, being in prison, having no information about the spouse)	4(26.7)	2(13.3)	
**Education**	Elementary/secondary education	4(26.7)	1(6.7)	0.33^**^
	High School/Diploma	8(53.3)	10(66.7)	
	University degree	3(20)	4(26.7)	
**Occupation**	Housewife	11(73.3)	10(66.7)	0.69^**^
	Office holder	4(26.7)	5(33.3)	

^*^Independent sample T-test

^**^Chi-square test

The data normality was measured by the Kolmogorov–Smirnov test, the result of which is listed in Table 3. According to *p*-value > 0.05, the data has a normal distribution (Table 3).

**Table 3:** Analyzing the normality of the pre-and post-intervention psychological well-being and its components

Variable	Pre-intervention		Post-intervention	
	T	*P*-value	T	*P*-value
**Autonomy**	0.88	0.42	0.98	0.28
**Environmental mastery**	1.11	0.16	0.73	0.65
**Personal growth**	0.81	0.51	1.50	0.20
**Purpose in life**	1.09	0.71	0.83	0.49
**Self-acceptance**	1.33	0.06	1.08	0.19
**Positive relationships with others**	0.95	0.32	0.91	0.36
**Psychological well-being**	0.65	0.78	0.63	0.82

Table 4 shows the descriptive indices (Mean ± SD) of psychological well-being and its components in the intervention and control groups. As seen in Table 4, in the pre-test stage, the mean ± SD of all psychological well-being components in the experimental and control groups are close to each other, but in the post-test stage, the scores of the intervention group have changed in the post-test stage compared to that of the ontrol group.

**Table 4:** Results of Mean ± SD of psychological well-being and its components in the intervention and control groups

Components	Pre-intervention		Post-intervention	
	**Intervention group**	**Control group**	**Intervention group**	**Control group**
	Mean ± SD	Mean ± SD	Mean ± SD	Mean ± SD
Autonomy	8.33 ± 1.71	8.80 ± 1.40	14.00 ± 2.03	8.40 ± 1.40
Environmental mastery	8.93 ± 1.57	8.33 ± 1.58	8.00 ± 1.64	8.80 ± 1.32
Personal growth	8.60 ± 1.40	8.53 ± 0.51	11.53 ± 1.50	8.80 ± 1.32
Purpose in life	11.73 ± 1.09	7.86 ± 1.18	11.53 ± 1.59	7.93 ± 0.88
Self-Acceptance	8.66 ± 1.54	4.60 ± 1.54	11.40 ± 1.91	4.33 ± 1.17
Positive relations with others	8.80 ± 1.26	8.86 ± 2.06	12.06 ± 2.60	8.73 ± 1.27
Psychological well-being	55.05 ± 8.57	46.98 ± 8.27	68.52 ± 11.27	46.99 ± 7.36

As depicted in Table 5, the results of the univariate covariance analysis of the mindfulness-based training group can be seen.

**Table 5:** Results of univariate covariance analysis of psychological well-being and its components in the mindfulness-based group

**Components**		**Sum of squares**	**Test statistics**	**Mean of squares**	**η**	**Power**	**Sig**
**Autonomy**	Group	203.47	73.35	203.47	0.731	1.00	0.001
	Pre-test	1.50	73.35	1.50	0.020	0.110	0.460
	Error	74.89		2.77			
**Environmental mastery**	Group	0.744	0.278	0.744	0.010	0.80	0.602
	Pre-test	1.079	0.403	1.079	0.015	0.094	0.531
	Error	72.255		2.676			
**Personal growth**	Group	53.949	45.075	53.949	0.625	1.00	0.001
	Pre-test	3.151	2.632	3.151	0.089	0.347	0.116
	Error	32.316		1.197			
**Purpose in life**	Group	97.044	48.786	97.044	0.644	1.00	0.001
	Pre-test	1.758	0.884	1.758	0.032	0.148	0.355
	Error	53.708		1.989			
**Self-acceptance**	Group	320.133	136.656	320.133	0.835	1.00	0.001
	Pre-test	21.949	9.370	21.949	0.258	0.839	0.005
	Error	63.251		2.343			
**Positive relationships with others**	Group	76.133	13.434	76.133	0.332	0.942	0.001
	Pre-test	1.657	0.292	1.657	0.01	0.082	0.593
	Error	153.010		5.667			
**Psychological well-being**	Group	1420.788	28.957	1420.788	0.517	0.999	0.001
	Pre-test	144.321	2.941	144.321	0.098	0.380	0.098
	Error	1324.746		49.065			

## Discussion

This research was performed pursuing the goal to develop the mindfulness-based training package and evaluating its impact on the psychological well-being of the female heads of the households covered by the welfare organization in Shiraz. Pursuant to the study extracted findings, the mindfulness-based educational intervention has promoted the psychological well-being of the female heads of the households of the above group. This way that over time and running the mindfulness-based educational intervention has brought about increased mean of psychological well-being and its components.

As the present study drawn results indicated, the educational intervention has led to an increase in the mean psychological well-being of the experimental group compared to the control group, implying the effectiveness of training in promoting the psychological well-being of the experimental group. This finding was consistent with the results of various studies, such as the ones conducted by Ajilchi et al. (2022) [25], Jones et al. (2022) [26], Zheng et al. (2022) [27], Monemiyan et al. (2021) [28]. In many cases, of female heads of households are not aware of the appropriate behavior in times of hardship and show an inappropriate reaction to it unintentionally, but with training based on mindfulness, women can improve their knowledge of how to deal with hardship. The improvement in women’s awareness of well-being can be attributed to the application of the mindfulness training.

In study done by Ajilchi et al. (2022) with the goal to find out the efffect of mindfulness-based training program on mental toughness and psychological well-being of female athletes, the result showed that mindfulness-based training program can be useful for psychological well-being [25]. Moreover, in the studies performed by Jones et al. (2022) [26] and Zheng et al. (2022) [27], the results were identical. These researchers reported that mindfulness-based intervention can improve psychological well-being and the quality of life [26, 27]. In another study carried out by Monemiyan et al. (2021), the finding was similar to that of our research [28].

Also, it can be claimed that the mindfulness components’ training results in a change in the beliefs of female heads of the households and a change in their life path. By benefiting from the training on mindfulness-based therapy, these women learned how to tackle tough and demanding life situations and give new meaning to their lives. In fact, mindfulness is to be aware of the present moment without judgment, such an awareness leads to the surrounding environment, to one's thoughts and feelings, without fixing anything or considering it good or bad [29].

The results of the current research revealed that after implementing the educational intervention, the mean of autonomy significantly increased in the intervention group compared with that of the control group, In general, performing health interventions when they are focused on the autonomy structure leads to the improvement of that structure in the trained person and makes the person gain a better attitude towards their skills and become more determined to put aside inappropriate attitudes. It seems that training mindfulness could effectively contribute to diminishing some outdated attitudes towards women, such as autonomy. This finding is consistent with the results of the studies by Altinyelken et al. (2020) [30], Bostock et al. (2019) [31], Rezaeian et al. (2019) [32]. Altinyelken et al. (2020) reported that mindfulness can work as a moderator for improving psychological well-being and by this kind of moderator and design intervention,we can boost psychological well-being [30]. Bostock et al. (2019) suggested that applying mindfulness meditation app can reduce work stress and promote well-being [31]. Moreover, the results were similar to that reported by Rezaeian et al. (2019).This researcher and his fellows reported that psychological empowerment can affect the well-being and quality of life of the women heading the household [32]. To justify this finding, we can state that by considering the components such as autonomy, personal growth, and self-mastery, mindfulness training teaches people to learn the due techniques to accept themselves without judging, live in the moment, and find a new insight into their life.

After the intervention, a sharp rise was seen in the personal growth aspect in the experimental group compared to the control group. It seems that the training increased the personal growth aspect and health beliefs of the trained women in the field of well-being, and this led to an increase in well-being behaviors in them compared to the control group. These findings are consistent with those of the study done by Lahtinen et al. (2023) targetting to discover the effects of app-based mindfulness practice on the well-being of university students and staff [33], and the research performed by da Silva et al. (2023) following the goal to determine the effectiveness of mindfulness-based training programs in reducing the psychological distress and promoting the well-being of medical students [34]. Mindfulness-based training acts as a meditation, it leads to an increase in hope, strength, flexibility, honesty, and self-acceptance in the person being trained, and finally, this person gets to know herself/himself better and tries to accept herself/himself without any judgment. Such an individual gains more control over herself/himself and learns to recognize all their weaknesses and forgive herself/himself.

In addition, after running the educational intervention, the environmental mastery in the experimental group got meaningfully higher compared to that of the control group. This finding was in agreement with the results found by Tang et al. (2019) [35], Kwon et al. (2020) [36], and Safari et al. (2020) [37]. Tang et al. (2019) targetting to discover the Promoting psychological well-being through an evidence-based mindfulness training program [35], and the research conducted by Safari et al. (2020) following the goal to determine the Efficacy of Mindfulness-Based Stress Reduction on Improvement of the Perceived Stress and Symptoms of Depression among Women-Headed Households [36], all these research support thie idea that mindfulness training program can improve psychological well-being.

Based on the results, the post-educational intervention resulted in the mean purpose in life significantly increasing in the intervention group compared with that of the control group, the findings which were consistent with those of the studies carried out by Taziki et al. (2022) [38], Bahreini et al. (2022) [39], Adelian et al. (2021) [40]. In interpreting this finding, it can be stated that implemmenting the training programs complying with the psychological well-being content and using suitable training strategies have resulted in the psychological well-being getting enhanced. Mindfulness-based training can make a person aware of herself/himself and help her/him predict and create a new way and approach in life.

In this study, education based on the mindfulness led to an increase in the the Self-acceptance of the Women of the experimental group compared to the control group. In this study, education based on the structure of perceived acceptance led to an increase in understanding of the importance of self-acceptance as a way to reduce anger, stress. Moreover, changes in acceptance across the intervention were correlated with session-specific changes in stress and mood, These findings are consistent with the studies of Anderson et al. (2023) [41], Laakso et al. (2023) [42], and Kramer et al. (2023) [43].

In this study, education based on the mindfulness led to a substantial rise in the average positive relationships with others of the women in the experimental group compared to the control group. In justification of this finding, it can be stated that in general, performing health-based interventions when focused on the need of female heads of the household leads to an improvement in this structure in the trained population and makes the person more aware positive relationships. These women know that having positive relationships with others makes them feel good, interact positively with others, consult with others about problems, share their emotions, and in addition, strengthen social relationships. These findings are in line with the results of Selič-Zupančič et al.’s study (2023) [44] and the study of Gallego et al. (2023) [45].

Lastly, education based on the mindfulness increased the well-being of the experimental group compared to the control group. In other words, the interventions focused on the mindfulness ultimately led to the change and improvement of the well-being of the women participating in this study, and this improvement can lead to the improvement of their mental health status, however like other studies, this study has strengths and limitations. One of the strengths of this study is the involvement of the researcher in executing the study, designing the educational intervention based on the pre-test results and employing face-to-face teaching methods, all group members actively participating, presenting the materials in the groups, and the participants having plenty of time to use the mentioned content. However, one of the study weaknesses is the impossibility behind generalizing the present results to other study populations.

## Conclusion

As the current study extracted results indicated, the mindfulness-based educational intervention led to an increase in the psychological well-being of the experimental group. Therefore, it is suggested to hire mindfulness-based intervention in implementing intervention programs for the women as the heads of the households. In the same way, through providing the required programs and services for preventing psychological problems at a lower cost by the government (under the insurance coverage of the services), the occurrence of such types of problems can be hindered. Similarly, considering the effect of educational intervention, the experts of health and psychology are proposed to recruit mindfulness-based intervention in their interventions and compare its results in all age and gender groups.

## Data Availability

The data supporting the findings of this study are available from [the corresponding author]; however, the availability of the data is subjected to restrictions for being applied because they are used under license for the current study, and thus, they are not publicly available. Anyway,the data can be acceesible if demanded from the authors and with the permission of [the corresponding author].
